# Whole transcriptome analysis and validation of metabolic pathways in subcutaneous adipose tissues during FGF21-induced weight loss in non-human primates

**DOI:** 10.1038/s41598-020-64170-6

**Published:** 2020-04-29

**Authors:** Sara A. Murray, Louise S. Dalbøge, Karalee Baquero, Christina A. Sanford, Ayesha Misquith, Aaron J. Mercer, Thomas H. Meek, Mette Guldbrandt, Birgitte Andersen, Paul Kievit, Kevin L. Grove, Burak Kutlu

**Affiliations:** 1 Novo Nordisk Research Center Seattle, Inc, Seattle, WA USA; 2 Gubra, Horsholm, Denmark; 3 Novo Nordisk A/S, Måløv, Denmark; 4 Division of Diabetes, Obesity & Metabolism, Oregon Primate National Research Center, Oregon Health Sciences University, Beaverton, OR USA

**Keywords:** Gene regulatory networks, Obesity, Drug development, Experimental models of disease, Preclinical research

## Abstract

Fibroblast growth factor 21 (FGF21) induces weight loss in mouse, monkey, and human studies. In mice, FGF21 is thought to cause weight loss by stimulating thermogenesis, but whether FGF21 increases energy expenditure (EE) in primates is unclear. Here, we explore the transcriptional response and gene networks active in adipose tissue of rhesus macaques following FGF21-induced weight loss. Genes related to thermogenesis responded inconsistently to FGF21 treatment and weight loss. However, expression of gene modules involved in triglyceride (TG) synthesis and adipogenesis decreased, and this was associated with greater weight loss. Conversely, expression of innate immune cell markers was increased post-treatment and was associated with greater weight loss. A lipogenesis gene module associated with weight loss was evaluated by testing the function of member genes in mice. Overexpression of NRG4 reduced weight gain in diet-induced obese mice, while overexpression of ANGPTL8 resulted in elevated TG levels in lean mice. These observations provide evidence for a shifting balance of lipid storage and metabolism due to FGF21-induced weight loss in the non-human primate model, and do not fully recapitulate increased EE seen in rodent and *in vitro* studies. These discrepancies may reflect inter-species differences or complex interplay of FGF21 activity and counter-regulatory mechanisms.

## Introduction

Fibroblast growth factor 21 (FGF21) is an endocrine regulator of lipid metabolism and energy homeostasis that has shown promise as a therapeutic against obesity and type 2 diabetes. In rodent and non-human primate (NHP) models, administration of FGF21 lowers blood glucose, plasma lipid levels, and body weight. In addition, data from several short-term clinical trials with FGF21 analogues confirm the strong effect on TG levels (up to 50% reduction) and, to a lesser degree, the reduction of body weight and blood glucose levels in humans^1–3^.

Though the metabolic effects of FGF21 treatment in primates are well documented, a clear understanding of the pathways by which FGF21 reduces body weight and improves plasma lipid profile in primates remains elusive. Several studies in rodent models suggest that FGF21 causes weight loss by acting upon adipocytes as well as the central nervous system (CNS) to induce browning and thermogenic pathways within adipose tissue (AT)^4–7^. Enhanced lipoprotein catabolism in both white and brown AT has also been proposed as the mechanism by which FGF21 lowers plasma TG levels in rodent models^8^. Additionally, recent studies in mice have indicated a role for innate immune signaling, supporting FGF21-mediated energy expenditure in adipose tissue^9,10^. Whether similar pathways are induced by FGF21 treatment in primates has not been fully explored.

Systems biology approaches using gene expression data can provide powerful insight into the signaling and regulatory pathways active during disease and drug responses. Weighted Gene Correlation Network Analysis (WGCNA) can be used to construct data-driven modules of genes whose expression may be coordinately regulated^11^. Changes in these gene modules can then be modeled against treatment groups or phenotypical parameters to gain understanding of the biological mechanisms driving disease pathology or drug activity. Transcriptomic studies have been used to gain understanding of the biological pathways active during drug treatment and weight loss^12–17^, but such an analysis has not been employed to understand the mechanisms of FGF21 activity in obese primates. Several gene expression studies in mice demonstrate increased expression of lipid transport and metabolic pathways in adipocytes in response to FGF21 treatment, reflecting elevated EE observed in oxygen consumption assays^12,13,18,19^. Additionally, numerous mouse and human studies have explored the transcriptomic response of AT to periods of caloric-restriction, reporting decreased expression of metabolic pathways associated with weight loss^14–17,20^. To date, there have been no reports detailing the full transcriptional response to FGF21 in any primate species, nor any attempts to infer gene expression networks active during FGF21 treatment in an obesity model.

We previously reported FGF21-induced weight loss in diet-induced obese (DIO) rhesus macaques^21^. The animals lost an average of 18% body weight over the course of the 12-week treatment, and regained weight to baseline levels during the 16-week washout period after treatment was stopped. Additionally, FGF21 treatment decreased plasma TG levels by 50%. Contrary to previous studies in NHP, there was no significant decrease in food intake during the treatment period, suggesting that increased EE may have played a role in the observed weight loss^21^. This intriguing result prompted us to further investigate metabolic pathways influenced by FGF21 at the transcriptional level in both subcutaneous AT and skeletal muscle. In the current report, we characterize, for the first time, the full transcriptional response to FGF21 treatment and weight loss in DIO NHP. In adipose tissue we observe transcriptional changes in adipogenesis, lipid synthesis, and neuronal signaling gene networks, as well as changes in immune cell markers and genes involved in thermogenesis that were associated with greater weight loss. Together these observations provide evidence for a shifting balance of lipid storage and metabolism related to FGF21 treatment and resulting weight loss in NHP that is distinct from previously described effects in rodent and *in vitro* models.

## Results

### FGF21 treatment induces transcriptional changes associated with weight loss in subcutaneous AT

In order to gain insight into metabolic changes that occur as a result of FGF21-induced weight loss, we examined gene expression in AT and skeletal muscle (SM) of DIO NHP undergoing treatment with FGF21. AT is a well-characterized site of increased EE in FGF21-treated mice, while SM, though not thought to be a direct target of FGF21, is capable of dramatically increasing EE in response to appropriate stimuli and may respond to secondary effects of FGF21 treatment. We obtained tissue punch biopsies of subcutaneous AT and gastrocnemius SM at three time points during the study: pre-treatment, week 12 at the end of treatment (“post-treatment”), and 16 weeks after treatment was stopped (“washout”), and performed RNA sequencing. We compared gene expression between time points to find transcriptional responses to FGF21 treatment. Additionally, variation in the amount of weight the animals lost over the course of the study [8–28%]^21^ allowed us to determine whether any gene expression changes were associated with greater weight loss.

**Figure 1:**
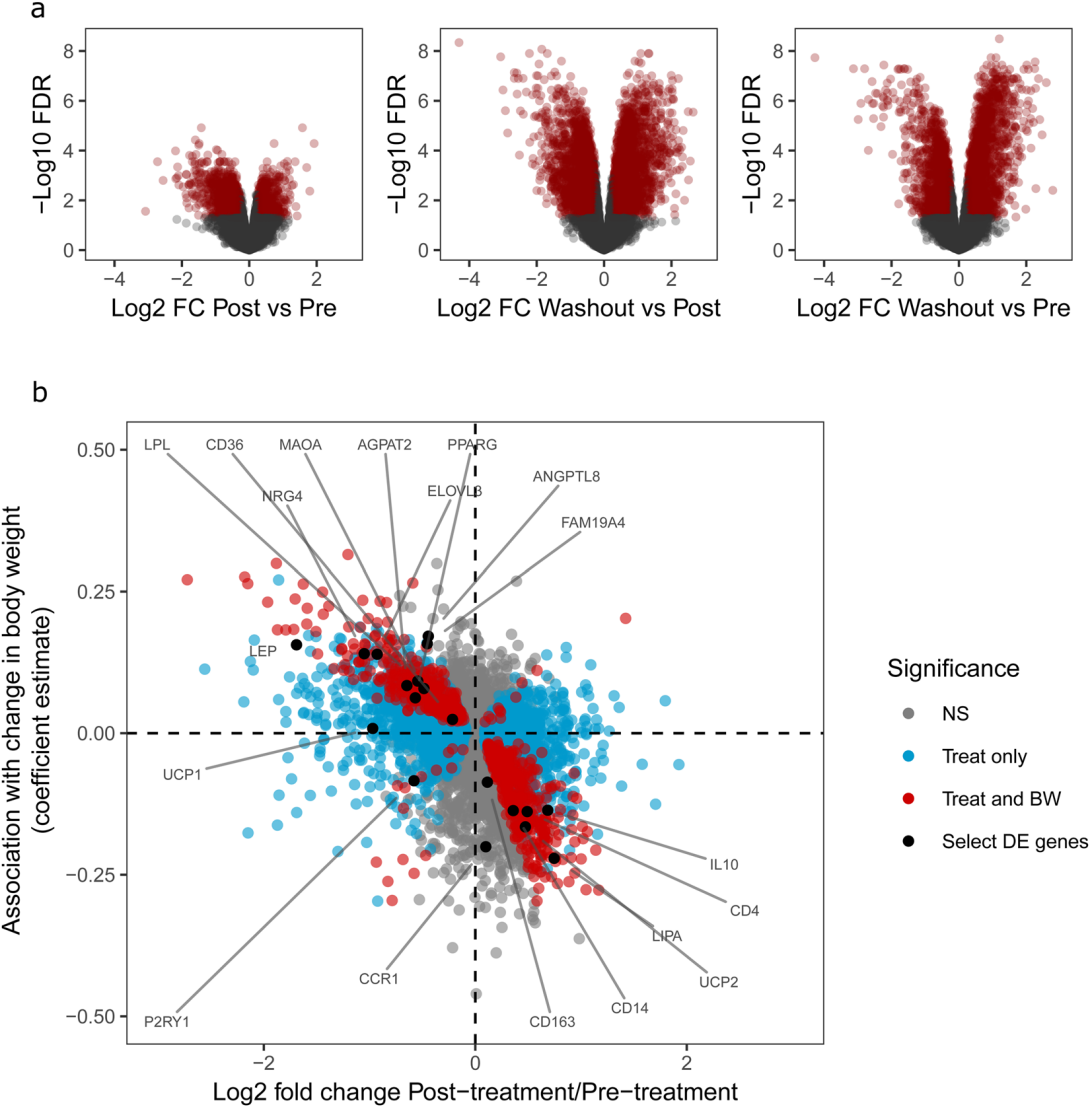
FGF21 treatment and weight loss associated transcriptional changes in AT. Gene expression in AT was measured by RNA sequencing. (**a**) Fold change and statistical significance of genes DE between treatment time points. Genes with fold change >0.26 or < −0.26 and FDR < 0.05 are highlighted in red. (**b**) Fold change of genes between pre- and post-treatment groups against the coefficient estimate for the association between change in gene expression and change in body weight, as determined by linear regression. Blue, significant DE genes in pre vs post comparison (as in A); red, genes with FDR < 0.25 in pre vs post that are also significantly associated with change in body weight, FDR < 0.25. Select genes of interest are labeled in black. n = 8–10 per time point in adipose tissue and n = 6–8 in skeletal muscle.

Data from AT revealed 2,005 differentially expressed (DE) transcripts between pre- and post-treatment time points (false discovery rate (FDR) < 0.05). Furthermore, the magnitude of change in 607 of those 2,005 genes was associated with the magnitude of weight loss (FDR < 0.25) (Fig. 1, Table 1, and supplemental fig. S1 and table S1). Pathway enrichment analysis of the top DE genes in the post- vs pre-treatment time points revealed that pathways relating to lipid transport and metabolism, glycolysis, protein translation, and cell cycle responded to FGF21 treatment. Top DE genes whose magnitude of change was also associated with increased weight loss showed enrichment for carbohydrate metabolism and endo-lysosomal pathways (supplemental table S2). Surprisingly, pathways related to browning of AT, such as ‘Function of brown adipose tissue’ from the Ingenuity Pathway Analysis (IPA) tool^22^, were not enriched amongst the top DE genes in either comparison. Likewise, there was no increase in the expression levels of the classic fat browning markers UCP1, CPT1B, and PPARGC1A at the post-treatment time point. In fact, UCP1 showed a significant decrease in expression post-treatment. We did not detect significant associations with increased weight loss for any of these markers.

**Table 1:** Summary of DEG numbers in AT and SM.

	Adipose tissue			Skeletal muscle			Significance criteria
	Post vs Pre	Wash vs Post	Wash vs Pre	Post vs Pre	Wash vs Post	Wash vs Pre	
Up	804	1840	2203	0	20	520	FDR < 0.05, LogFC > 0.26 or < −0.26
Down	1200	1900	2284	0	3	474	
Total	2005	3740	4487	0	23	994	
Increased expression with greater BW loss	299	NA	NA	0	NA	NA	LogFC > 0.26 or < −0.26 for Pre vs Post, and FDR < 0.25 for both Pre vs Post and association with change in BW
Decreased expression with greater BW loss	308	NA	NA	0	NA	NA	

In each tissue, the number of significant DEG (FDR < 0.05) in each time point comparison is listed. The number of genes with an FDR < 0.25 between post- and pre-treatment time points and whose change in expression was also associated with change in body weight are summarized in the bottom two rows. n = 8–10 per time point for AT and n = 6–8 for SM.

Unlike the response in AT, in SM we observed a high level of intra-group variation in the transcriptional response, which resulted in a lack of statistically significant DE genes between pre- and post-treatment time points (FDR < 0.05), though we observed 994 DE genes at the washout time point compared to pre-treatment (Table 1, supplemental fig. S1, and table S1). Because of this high, unexplained variability in the muscle, we investigated the response to FGF21 in the AT data only.

### Network analysis reveals several metabolism related gene modules in AT and SM

**Figure 2:**
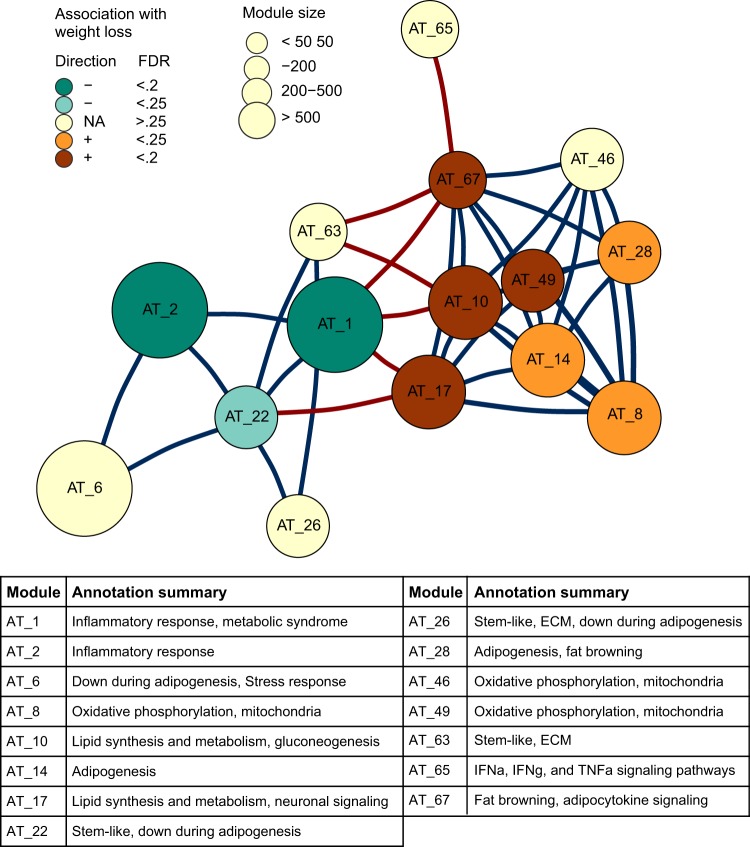
Correlation network analysis reveals several metabolism related gene modules in AT. WGCNA was used to group genes with coordinated expression into modules within AT. The relationship between select modules annotated with metabolic disease-related pathways is shown in a correlation network. Nodes are gene modules and edges represent the Spearman correlation between module eigengenes (blue, positive; red, negative). Only correlations stronger than r = 0.6 or −0.6 are shown. Node size depicts the number of genes in the module. Node color represents strength and direction of association of change in module expression with change in body weight. Bottom, short descriptions of modules included in the network, based on their overlap with known biological pathways (supplemental table S3). ECM, extracellular matrix.

To investigate how metabolic and other signaling pathways responded to FGF21-induced weight loss, we applied WGCNA^11^ to create a network of data-driven gene modules within each tissue transcriptome (Fig. 2 and supplemental table S3). This resulted in 71 gene modules in AT, renamed AT_1 to 71 from largest to smallest module. The modules were then annotated by assessing their overlap with gene ontology or literature-derived pathways using a hypergeometric test (supplemental table S4).

Fifteen of the AT modules were significantly associated with metabolic disease-related pathways (supplemental table S4a). We examined the relationships between these 15 modules by constructing a network using the pairwise correlations between module eigengenes (Fig. 2). The network revealed two major clusters of positively correlated modules; one cluster containing modules related to adipogenesis, fat browning, and lipid metabolism (such as AT_10, AT_17, AT_28, and AT_46), was negatively correlated with a second cluster containing modules related to immune signaling, extracellular matrix pathways, and stem-ness (including AT_1, AT_22, AT_26, AT_63), suggesting FGF21 stimulates opposing responses in these pathways.

Despite the inconsistent response to FGF21 in the skeletal muscle, the variation in the SM expression data provides a wealth of information on the interaction and association of genes into biological pathways. Thus we again used WGCNA to construct and analyze a separate correlation network within the skeletal muscle. Of the resulting 44 SM gene modules, 14 were significantly associated with pathways related to metabolism or metabolic disease, such as oxidative phosphorylation, insulin signaling, and stress response (supplemental table S4b). A correlation network analysis between module eigengenes revealed a glycolysis and gluconeogenesis module (SM_3) as a central hub, positively correlated with insulin signaling (SM_8), oxidative phosphorylation (SM_10), and stress response modules (SM_5, SM_9), and negatively correlated with a vasculogenesis and extracellular matrix module (SM_1) (supplemental fig. S2). Also of note was that a module related to myogenesis (SM_40) had no strong connections to any other metabolism-related modules (supplemental fig. S2). Similar to the results of differential expression analysis on individual genes in the SM, none of the SM modules responded to FGF21-induced weight loss in a consistent or statistically significant manner (supplemental table S5).

### FGF21-induced weight loss modulates expression of adipogenesis, browning, and lipid synthesis and storage modules in AT

**Figure 3:**
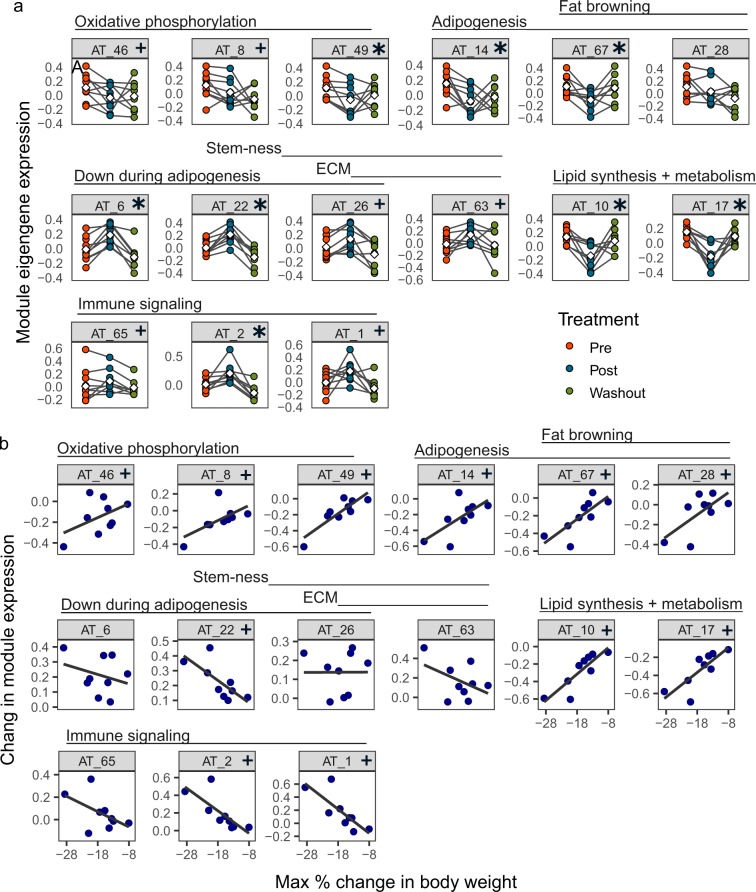
FGF21-induced weight loss modulates adipogenesis, fat browning, lipid metabolism, and inflammatory pathways. (**a**) Expression levels of metabolism and immune-related gene module eigengenes by time point and (**b**) the association between change in module expression between pre- and post-treatment and decrease in body weight. White diamonds indicate mean expression at each time point. * FDR < 0.1, + FDR < 0.25 between pre- and post-treatment groups by Wilcoxon paired test (n = 8–10) (**a**), or by linear regression between change in module expression and change in body weight (n = 9) (**b**).

We investigated how FGF21 treatment and resulting weight loss influenced modules related to energy expenditure and adipogenesis in AT by comparing their expression levels between time points. We also tested whether changes in any of the modules correlated with the magnitude of weight loss observed (supplemental table S5). We observed a mixed response in three gene modules that had significantly overlap with oxidative phosphorylation or mitochondrial function gene sets^23,24^ (AT_46, AT_8, and AT_49) (Fig. 3a). Expression of all three modules was decreased at the post-treatment time point, and decreased expression of modules AT_8 and AT_49 was associated with greater body weight loss (Fig. 3b). Additionally, expression levels of modules related to triglyceride and fatty acid biosynthesis (AT_10 and AT_17) were decreased at the post-treatment time point (Fig. 3a). This decrease was associated with greater weight loss for both modules (Fig. 3b).

The expression of modules annotated with adipogenesis (AT_14, AT_28, AT_67) and fat browning pathways (AT_28, AT_67) was decreased after 12 weeks of FGF21 treatment and resulting weight loss, generally returning to baseline levels during the washout period (Fig. 3a). Decreased expression of these modules was associated with greater loss of bodyweight (Fig. 3b). Modules AT_6, AT_22, AT_26, and AT_63 contain genes expressed by stem cells^25^, during extracellular matrix remodeling, and genes known to exhibit decreased expression during adipogenesis^26^. Expression of these modules was elevated at the post-treatment time point (Fig. 3a). Additionally, increased expression of the stem cell-like module AT_22 was associated with greater decrease in body weight (Fig. 3b).

### FGF21-induced weight loss changes the immune profile of adipose tissue

Recent studies have indicated a role for innate immune cells and signaling pathways in supporting FGF21-mediated EE in mice^9,10^, thus we examined the response of three gene modules annotated as immune-related: AT_1, AT_2, and AT_65. Interferon signaling pathways (AT_65) did not change with treatment or weight loss. Conversely, expression of module AT_2, which is associated with phagocytic endolysosomal and TNF signaling pathways, increased during FGF21-induced weight loss, and increased expression was associated with greater weight loss (Fig. 3a,b). Module AT_1, which contained genes associated with type 2 signaling and complement pathway, as well as canonical markers for several immune cell populations^27^, was similarly increased at the post-treatment time point, and increased expression was associated with greater weight loss. Moreover, individual markers of type 2 signaling, natural killer T cells, and macrophages showed elevated expression during FGF21 treatment, and for many of these genes, this change was associated with increased weight loss (supplemental fig. S3a).

### Correlation of genes involved in neuronal signaling, thermogenesis, and immune pathways

**Figure 4:**
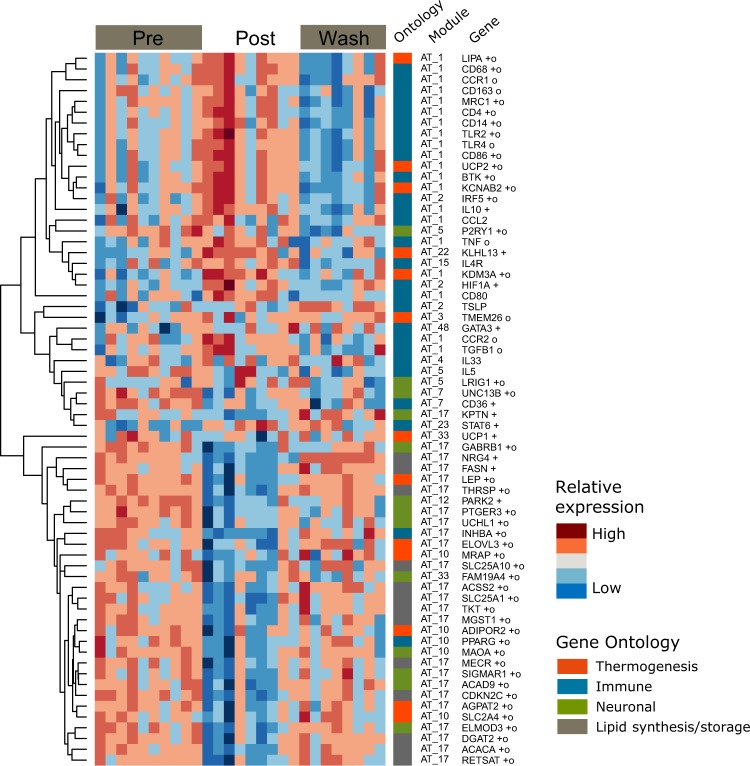
Divergent responses of genes involved in thermogenesis. (**A**) Gene expression heat map depicting select genes related to metabolic and inflammatory pathways. Each column depicts one sample from one treatment time point and animals appear in the same order within each time point. Each row represents one gene, and rows are hierarchically clustered to group genes with similar expression patterns. The module and the general ontological category each gene is a member of is depicted in columns between the heat map and gene names. n = 8–10 per group. +, genes with significantly different expression between pre and post time points (FDR < 0.05); o, genes whose changes in expression were significantly associated with decreased body weight (FDR < 0.25).

The lipogenesis module (AT_17) contains genes involved in triglyceride and fatty acid biosynthesis, as well as several genes involved in neuronal signaling. The sympathetic nervous system likely plays a role in regulating the balance between lipolysis for oxidative phosphorylation and lipid uptake for storage^5,7,28,29^. Because decreased expression of AT_17 was associated with greater weight loss, we further investigated transcriptomic changes in this system by examining the response of genes involved in norepinephrine signaling, neurotransmitter release, and innervation compared to genes that regulate thermogenesis^22,30^ (supplemental table S6). We observed an intriguing pattern of expression and correlation amongst genes involved in neuronal signaling, lipogenesis, thermogenesis, and immune pathways that were significantly changed by FGF21 treatment and/or associated with greater weight loss (Fig. 4 and supplemental fig. S3). A hierarchical clustering of these genes emphasizes the divergent responses of genes associated with thermogenesis. Perhaps unsurprisingly, then, genes related to browning and thermogenesis were spread across several of the data-driven modules. In addition to membership in the AT_28 and AT_67 modules annotated as ‘fat browning,’ several genes that have been shown to impact thermogenesis (KCNAB2, KDM3A, LIPA, UCP2)^22^ were contained in the AT_1 immune module. Expression of these genes increased with treatment, along with several pro- and anti-inflammatory immune cell markers and signaling genes. Conversely, other genes that support thermogenesis, lipogenesis, and neuronal signaling (largely contained in the AT_17 module) were decreased at the post-treatment time point.

### *In vivo* validation of lipogenesis module members

Given its association with increased weight loss and notable collection of neuronal and TG synthesis genes, we hypothesized that pathways in the AT_17 lipogenesis module may play a role in decreasing fat storage during FGF21-induced weight loss. To test this idea, we selected three genes—ANGPTL8, NRG4, and FAM19A4—from this module to evaluate independently for functional *in vivo* effects. These three genes encode secreted proteins with evidence of involvement in metabolic pathways, and thus may recapitulate some of the effects of FGF21 treatment we observed in NHP. We overexpressed each protein in separate experiments using hydrodynamic gene delivery^31,32^ in lean or growing DIO mice and measured the effects on body weight and plasma triglyceride levels. In all three studies, the positive controls (Exendin-4 or FGF21) were used to demonstrate that the plasmid delivery and expression worked as expected.

**Figure 5:**
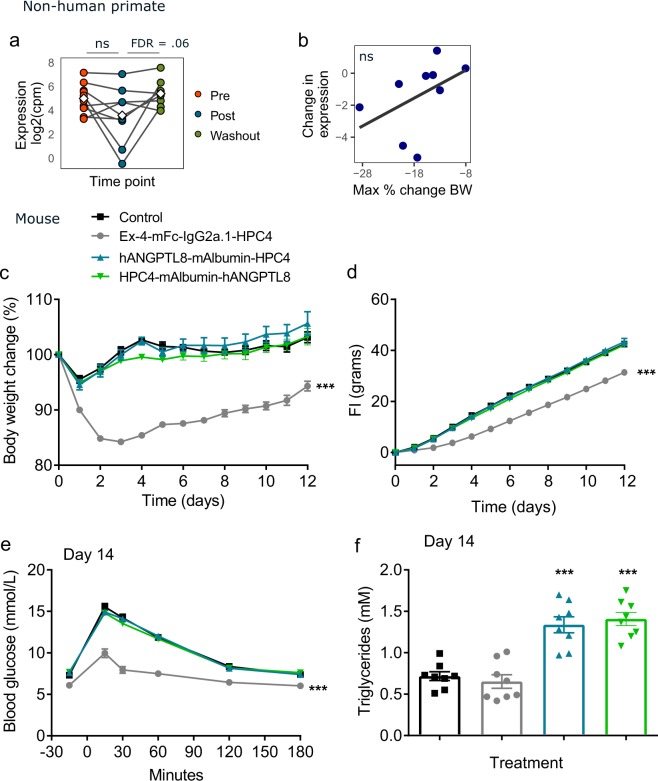
Overexpression of ANGPTL8 increased plasma triglyceride levels. ANGPTL8 was overexpressed in mice by hydrodynamic gene delivery. (**a**) Expression levels and (**b**) association with change in body weight in FGF21-treated NHP. (**c-f**) Mice were given empty plasmid, or plasmid encoding Exendin-4, N-terminal or C-terminal mouse albumin-fused human ANGPTL8 sequence. (**c**) Change in body weight and (**d**) cumulative food intake over time. (**e**) Blood glucose after glucose tolerance test and (**f**) plasma triglycerides at day 14. NS non-significant; (**a-b**) + FDR < 0.25 by linear model; (**c-f**) Data are displayed as mean plus standard error, n = 7–8 per group, *p < 0.05, **p < 0.01, ***p < 0.001 by one-way ANOVA with Dunnett’s post-hoc test.

In FGF21-treated NHP, ANGPTL8 expression was significantly increased during the washout period and exhibited a non-significant trend toward association with increased weight loss (Fig. 5a,b). ANGPTL8 is increased in humans during refeeding after fasting and is thought to play a role in regulating lipid metabolism levels^33–35^. Indeed, in our hands, overexpression of ANGPTL8 in lean mice significantly increased plasma triglyceride levels, but did not have an effect on body weight. Overexpression of the positive control Exendin-4 (a GLP1 receptor agonist) lowered body weight and food intake as expected (Fig. 5c–f).

**Figure 6:**
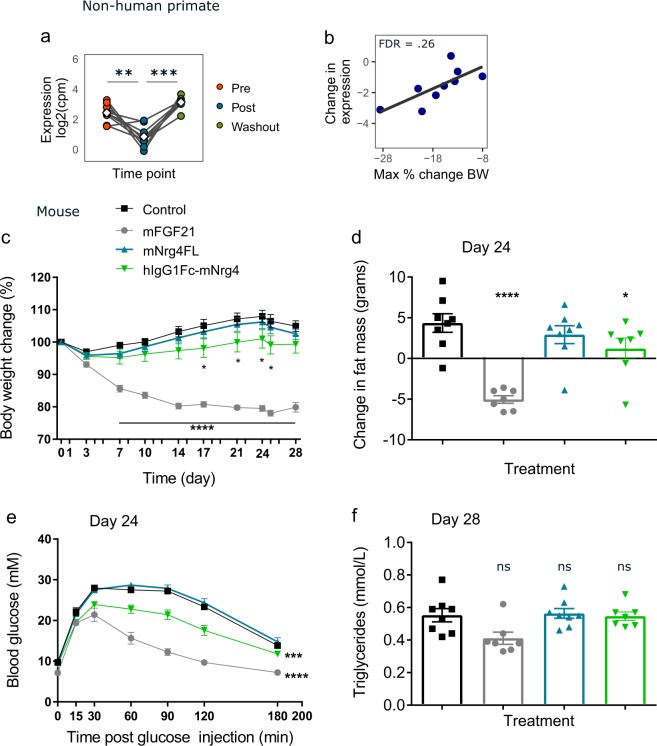
Overexpression of NRG4 reduced weight gain in DIO mice. NRG4 was overexpressed in mice by hydrodynamic gene delivery. (**a**) Expression levels and (**b**) association with change in body weight in FGF21-treated NHP. (**c**–**f**) Mice were given empty plasmid, or plasmid encoding mouse FGF21, native mouse Nrg4, or mouse Nrg4 fused to human IgG1Fc. (**c**) Change in body weight over time. (**d**) Change in fat mass from day 0 to day 24. (**e**) Blood glucose after glucose tolerance test and (**f**) plasma triglycerides at day 14. NS non-significant; (**a,b**) ** FDR < 0.01, ***FDR < 0.001 by linear model; (**c**–**f**) Data are displayed as mean plus standard error, n = 7–8 per group, *p < 0.05, **p < 0.01, ***p < 0.001 by one-way ANOVA with Dunnett’s post-hoc test.

NRG4 expression was decreased during FGF21-induced weight loss in NHP and trended toward association with greater weight loss, just missing our cutoff for statistical significance at an FDR of 0.26 (Fig. 6a,b). High levels of NRG4 have been detected in brown AT and increased expression of NRG4 has been reported in response to cold stimulation^36^. In light of this, we hypothesized that decreased expression of NRG4 during our study was a secondary response to weight loss rather than a direct response to FGF21 treatment, and that overexpression may inhibit weight gain. Indeed, overexpression of NRG4 moderately reduced weight gain in growing DIO mice (Fig. 6c), which appeared to be due to prevention of fat mass accumulation (Fig. 6d). The FGF21 positive control reduced body weight, indicating successful expression of the plasmid upon HGD. NRG4 overexpression had no effect on blood glucose, plasma TG levels, or food intake (Fig. 6e,f and not shown).

We found FAM19A4 to be highly expressed in AT in NHP, and decreased expression levels post-treatment were associated with greater weight loss (supplemental fig. S4a,b). Because FAM19A4 is a secreted protein and is expressed by neurons in several areas of the brain^37–39^, we hypothesized that FAM19A4 may signal between AT and the brain to modulate lipid handling or EE pathways. However, overexpression of FAM19A4 did not influence body weight, food intake, blood glucose, or plasma TG levels in lean mice, even though the positive control, Exendin-4, indicated that the HGD system was working properly (supplemental fig. S4c–f). We then investigated whether FAM19A4 may act directly in the brain by treating lean animals centrally with FAM19A4 protein. Consistent with the lack of response from peripheral expression, we did not observe any effects on food intake, brown AT temperature, or body weight (supplemental fig. S4g–i).

**Figure 7:**
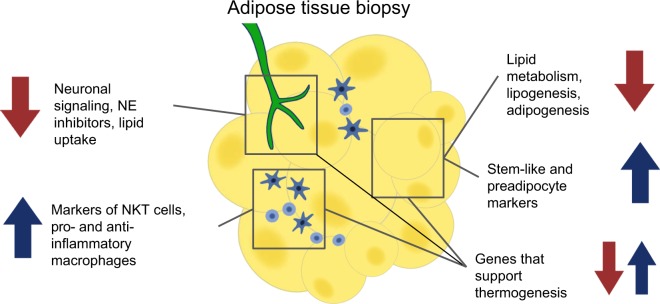
Summary of transcriptomic changes in AT in response to FGF21-induced weight loss. FGF21 treatment and resulting weight loss in NHP induced transcriptional changes in several metabolic pathways in AT, as measured by RNA sequencing of tissue biopsy punches. As a biopsy punch of adipose tissue contains a mixture of cell types, some of the transcriptional changes observed may originate from innervating neurons (green), immune cells (blue), or adipocytes (yellow). Blue arrows signify pathways or genes that were increased post-treatment compared to pre-treatment, and whose increase was associated with greater weight loss. Red arrows signify pathways or genes that were decreased at the post-treatment time point and whose increase was associated with greater weight loss.

In summary, at the end of a 12 week period of FGF21-induced weight loss, we observe transcriptional changes in several pathways related to metabolism and fat storage in adipose tissue. Figure 7 depicts our current model of the *in vivo* actions of FGF21 at the transcriptional level in primates. Genes related to thermogenesis responded inconsistently to FGF21-induced weight loss, some increasing and some decreasing by the post-treatment time point. Decreased expression of genes and modules involved in neuronal signaling, triglyceride (TG) synthesis, and adipogenesis was associated with greater weight loss, as was increased expression of immune genes and modules.

## Discussion

In the current report, we have examined the effects of FGF21 in two tissues at the mRNA level and investigated associations between changes in metabolic pathway expression and the degree of resulting weight loss. Our study differs from prior work in that it is the first to use a transcriptomic, systems biology-based approach to understand FGF21 activity in NHP. Moreover, it is the first attempt to explain the variability in weight loss across individuals in connection with variation in gene expression changes, using a primate model that more closely reflects the large inter-individual variability in humans.

Our results demonstrate the complexity of metabolic changes associated with fluctuations in body weight and energy balance, induced by artificially increasing levels of a single secreted factor. The response to FGF21 treatment measured in subcutaneous AT revealed shifting activity across adipogenesis, lipid metabolism, neuronal signaling, and immune signaling pathways. Together these changes provide mixed evidence in support of increased EE as a mechanism of FGF21-induced weight loss in the NHP model and may represent the complex interplay of the primary effects of FGF21 and the secondary effects of weight loss.

The animals in the present study did not significantly lower their food intake during the treatment period;^21^ thus we postulated that increased EE may have been responsible for the observed weight loss. In the transcriptional data, gene modules annotated with fatty acid oxidation or mitochondrial activity exhibited a moderate decrease in expression in response to 12 weeks of FGF21 treatment. This is in contrast to increased EE observed in FGF21-treated rodents and *in vitro* adipocyte models. However, a decrease of lipid metabolism in response to a period of significant weight loss is well documented — the body downregulates mechanisms that facilitate EE to avoid starvation^40^ — thus our observations may be linked to weight loss and not necessarily FGF21 activity. In support of this, numerous studies on the response of AT to weight loss induced by caloric restriction have observed decreased expression of genes involved in lipid handling and metabolism^14,15,17,20^.

Intriguingly, we observed divergent responses amongst genes and gene modules related to thermogenesis. In addition to decreased expression of modules related to fat browning, four individual genes that promote thermogenesis (LEP, PPARG, SLC2A4 and MRAP)^41,42^ were decreased at the post-treatment time point and decreased expression was associated with greater weight loss. Conversely, decreased expression of ADIPOR2 (adiponectin receptor 2), which has been reported to downregulate thermogenesis^43^, and increased expression of genes that have been reported to support thermogenesis (LIPA, KDM3A, KCNAB2, and UCP2)^44–48^ were associated with greater weight loss. These observations suggest that mechanisms of EE in NHP may not be as tightly coordinated as in rodents, or that FGF21 may influence a broader array of pathways in NHP, though FGF21-induced thermogenesis in rodents does not always involve canonical markers like UCP1^49^. At ambient temperature mice need to constantly defend their body temperature, increasing overall EE by activating brown fat^50^, which is not as critical in larger mammals due to their smaller surface-to-volume ratio. It is also likely that the dynamics of FGF21 action differ between rodents and primates, and it is possible that an earlier time point during treatment would have revealed a different hierarchy of pathways associated with weight loss.

In the current study, we observed that numerous genes involved in neuronal signaling were decreased as a result of FGF21-induced weight loss. Several of these have been shown to inhibit norepinephrine signaling and norepinephrine-mediated fat browning (P2RY1, MAOA, PTGER3, SIGMAR1)^51–55^, and in the present study, decreased expression was associated with greater weight loss. Several previous studies suggest that the balance between lipid storage and lipolysis for oxidative phosphorylation is regulated in part by sympathetic tone, mediated by norepinephrine signaling^5,7,28,29^. Supporting this, we observed that expression of gene modules related to adipogenesis (AT_14, AT_28) and lipogenesis (AT_17) were decreased post-treatment and associated with increased weight loss. Moreover, genes involved in promoting lipid uptake and storage (PARK2, KTPN2, CD36)^56–58^ also exhibited decreased expression post-treatment and were associated with greater weight loss. Additionally, we see an increase in markers of stem-like and pre-adipocyte cells, as well as extracellular matrix genes, suggesting the AT is likely undergoing shifts in cell type composition and structure. Taken together, these results may suggest FGF21 has shifted sympathetic nervous signaling away from induction of fat storage, which could offer a partial explanation for the animals’ weight loss.

Mounting evidence indicates the immune system interacts with metabolic pathways at multiple levels^59^. In the current study, we observed transcriptional evidence of a shifting immune cell populations and milieu in NHP experiencing FGF21-induced weight loss. Increased expression of IL10, TGFB1, and IL4R and their association with greater weight loss reflects previous studies that have demonstrated a role for type 2 signaling in mediating the recruitment and polarization of innate immune cells to facilitate fat browning and thermogenesis^9,10,12^. Also in line with previous reports, markers of inflammatory macrophages (CD86, CD80, CD68, TLR2, and TLR4), and anti-inflammatory or ‘alternatively activated’ macrophages (CD163, MRC1/CD206, IL10, and TGFB1) were associated with greater weight loss, suggesting the presence of a beneficial, mixed population of macrophages in the AT^14,60–62^. Local inflammation and influx of phagocytic cells may help remodel AT and buffer excess lipids during weight loss^63,64^.

This shift in immune cell markers could reflect a return to an immune landscape associated with healthy, functional AT. Intriguingly, expression of the AT_1 immune module is negatively correlated with expression of module AT_17, which contains genes involved in triglyceride and fatty acid synthesis as well as neuronal signaling. This may indicate that immune, neuronal, and lipogenesis pathways are acting in concert during FGF21 treatment, perhaps shifting the balance between fat storage and EE in the NHP model by influencing sympathetic nervous tone and the immune landscape of adipose tissue.

Validating some of our findings in the transcriptional data, differentially expressed genes related to lipid handling such as ANGPTL8 and NRG4 affected triglyceride levels and bodyweight when overexpressed independently in a mouse model. These results implicate ANGPTL8 as a possible mediator of FGF21’s lipid-lowering role, and suggest NRG4 may decrease as a secondary response to weight loss in order to prevent further reductions of adipocyte mass. Supporting this, previous studies have implicated NRG4 as a negative regulator of lipogenesis^65^.

Our results from the NRG4 and FAM19A4 mouse experiments reflect a broader truth that the transcriptional data do not provide any directional information, and we are unable to draw conclusions about whether changes in certain pathways are driving changes in others. As this study was based on cross-sectional snap-shots of gene expression at points in time before weight loss began and after weight loss was achieved, we cannot determine which changes were primarily due to FGF21 activity and which might be secondary responses to weight loss. Likewise, though we see a large number of DEG at the washout time point, we are unable to tease apart changes that may be long term effects of FGF21 treatment and those that may be due to having experienced significant weight loss and regain. Additionally, as we previously observed increases in protein levels of adiponectin, T4, and IGFBP2 and decreases in IGF-1 and myostatin at the post-treatment time point in these animals^21^, hormones such as these affected by FGF21 treatment and/or weight loss may also be influencing gene expression levels. To truly understand the causal hierarchy of pathway interactions and regulation, constant measurements across multiple energy expenditure and energy storage systems would be necessary.

While we initially hypothesized that skeletal muscle could be a site of increased EE in response to FGF21 activity, the large inter-individual variation in transcriptional changes in SM obscured our ability to draw conclusions about its role in FGF21-mediated weight loss. The variation we observed could indicate that skeletal muscle tissue does not respond in a consistent manner when animals are treated with FGF21. Another possible explanation is that an inconsistent mix of muscle fiber types (or other cell types) may have been obtained during the blind biopsy-punch procedure used to sample the tissue; red and white muscle fibers may respond differently to FGF21 treatment, as there is evidence they respond differently to cold exposure^66^.

In summary, we have explored the transcriptional response of subcutaneous adipose tissue to a 12-week course of FGF21 treatment and resulting weight loss in a cohort of diet-induced obese NHP. In the mouse model, numerous studies point to increased EE as a driving mechanism behind FGF21-induced weight loss^12,14,18,19^, but our results suggest that a direct increase in core EE mechanisms may not be a central component of FGF21 activity in primates. We observe what appears to be an overall pattern of decreased activity of lipid metabolism pathways at the end of the 12-week treatment and weight loss period. However, this pattern is peppered with contradictory increases in expression of several individual genes that promote thermogenesis and complicated by the observed changes in neuronal and immune pathways that have been shown to support thermogenesis. Taken together, our results may suggest that FGF21 does not act on all energy expenditure mechanisms equally, or potentially that even in the face of compensatory mechanisms arising due to weight loss, FGF21 is still acting on pathways that support fat browning and thermogenesis to drive weight loss.

Network analysis approaches using models that closely reflect the great diversity in human populations, such as described here, can begin to explain the variability in metabolic responses between species as well as across populations. A systems biology-based investigation of the effects of drug treatment and resulting weight loss allowed us to aggregate thousands of gene expression changes into a network of metabolism-related biological pathways in tissues relevant to obesity. Using a data-driven approach to identify gene modules in combination with published metabolic gene ontology pathways grants us access to novel insight into the activity of FGF21 in a model that more closely mirrors human patients. Many of our data-driven gene modules closely recapitulated well-established molecular pathways, such as immune signaling, adipogenesis, and lipid metabolism, lending validation to our methodology and suggesting at least some commonality between primate and rodent models of weight loss. Interestingly, several gene modules not discussed here, whose change in expression was associated with treatment or with greater weight loss, such as AT_58, AT_33, and AT_56, were not closely related to characterized pathways. These modules may represent interspecies differences in transcriptional regulation or novel associations between molecular pathways that arise during FGF21-induced weight loss. Further validation of these modules in larger NHP or human studies can be employed to elucidate master regulators of metabolic pathways and to gain insight into variability in weight loss mechanisms. Any opportunity to continue this avenue of research using clinical biopsies of fat and muscle over time would likely be hugely valuable to therapeutic advances in disease treatment.

## Methods

### Rhesus macaques

The cohort of rhesus macaques (Macaca mulatta) and their treatment with FGF21 have been described previously^21^. Briefly, 10 obese, non-diabetic rhesus macaques (5 males and 5 ovariectomized females), age 11–22 years old with bodyweights ranging from 8.3 to 21.1 kg were used in this study. The animals were maintained on a high-fat diet for at least 2 years prior to the start of the study. Animals were pair-housed (one male and one female) and had access to shelves and changeable plastic toys. The study was approved by the Novo Nordisk ethical council and all animal care and procedures were performed according to the Institutional Animal Care and Use Committee (IACUC) at the Oregon National Primate Research Center at Oregon Health Sciences University, as well as Novo Nordisk internal standards for non-human primate studies.

### Diet

NHP were fed a high-fat, high-sucrose diet (Diet 5L0P, 36.6% calories from fat, 45% from carbohydrates, and 18.4% from protein; cholesterol 612 ppm) LabDiet, Richmond, IN. Food was provided twice daily, and animals received a calorie-dense peanut butter treat, as well as additional enrichment (vegetables, fruit, or popcorn) every day.

### FGF21 protein

Production of native FGF21 was carried out at Novo Nordisk A/S sites in Denmark and China by methods described in patent number WO2016102562. Briefly, mature human FGF21 was expressed in inclusion bodies of *E. coli* BL21(DE3) cells, purified by standard chromatographical techniques, and transferred to phosphate buffered saline. The FGF21 protein carries an N-terminal methionine added by the *E. coli* expression host. The identity, purity, and stability of the FGF21 protein were confirmed by a variety of chemical and biophysical techniques.

### Dosing

Animals were treated by subcutaneous injection once daily for 12 weeks with increasing doses of native human FGF21, starting at a dose of 10 ug/kg/day, followed by 30, 100, 300, and 1,000 ug/kg/day over the first 6 weeks. Nine of 10 animals were maintained on 1,000 ug/kg/day for the last 6 weeks, and one animal was held at 300 ug/kg/day due to extensive weight loss. The treatment period was followed by a 16-week washout period.

### Phenotypical analysis

Phenotypical measurements (body weight, plasma triglyceride levels) were analyzed by one-way ANOVA with repeated measures using Prism (GraphPad Software, La Jolla, CA) as previously described^21^.

### Tissue biopsies for gene expression analysis

Subcutaneous abdomen adipose tissue and gastrocnemius skeletal muscle biopsies were collected from the animals under isoflurane anesthesia at baseline, after 12 weeks of treatment, and after 16 weeks of washout. The tissue biopsies were snap frozen in liquid nitrogen and stored at -80 degrees.

### RNA isolation

Samples were thawed and homogenized in Trizol with one 5 mm stainless steel ball using a TissueLyser II (Qiagen, Hilden, Germany). RNA was extracted using chloroform and a Qiagen RNeasy Mini Kit following the manufacturer’s instructions. RNA concentration and quality was measured on a Nanodrop and Bioanalyzer and samples with a 260/280 ratio of> 1.8 and RIN > 8 were shipped on dry ice for sequencing. We were unable to isolate quality RNA from 2 adipose tissue and 6 skeletal muscle samples and these were therefore not included in the RNAseq analysis.

### RNA sequencing and alignment

Sequencing was performed by Covance Genomics Lab, Redmond, WA. cDNA libraries were prepared with Illumina TruSeq stranded mRNA kit and sequenced on an Illumina HiSeq. 2000 using paired-end, 50 nucleotide reads, with a read depth of 20–30 million reads per sample.

Sequenced reads were aligned by STAR^67^ to the MacaM Rhesus macaque reference genome^68^. For use in downstream gene ontology and pathway analysis, macaque genes were matched to the closest human homolog, defined as a gene with a symbol that matched the macaque symbol and had a canonical Uniprot entry. One adipose tissue sample failed sequencing and alignment and was excluded from the analysis.

### Sample library QC

Samples that had at least 75% reads uniquely mapped to the genome, and at least 10 million total mapped reads were included in the analysis. Three skeletal muscle samples that fell below these cutoffs were excluded.

### Sample identity check

Sample identity was confirmed by two methods. First, the median expression of genes known to be highly expressed in or specific to skeletal muscle was plotted against the median expression of genes known to be highly expressed in or specific to adipose tissue (Human Protein Atlas)^69^. Samples fell into two clusters; AT samples expressed high levels of adipose genes and low levels of skeletal muscle genes, whereas SM samples expressed high levels of skeletal muscle genes and low to medium levels of adipose genes (supplemental figure S5a and b).

In a second step, single nucleotide polymorphism (SNP) variants were compared between sample libraries to confirm that samples from the same animal did indeed come from the same animal. Because there was no existing list of genomic variants available for the MacaM rhesus macaque genome version, a list of SNPs was compiled from the indexed BAM files of all sequenced samples using samtools mpileup and filtered to high confidence SNPs using bcftools filter. The list was further trimmed to include 5,049 SNPs that were expressed at a read depth of greater than 100 and less than 400 in all samples to include variants that were highly expressed regardless of tissue type. Variants from each sample were compared to all other samples using BAM-matcher^70^ with the Varscan option^71^. Fraction of SNPs in common between each sample pair were plotted in a hierarchically clustered heatmap (heatmap.plus R package) to visualize similarity (supplemental figure S5c). Samples across time points and tissue from the same animal clustered together with a fraction in common above 90%, while samples from different animals had a fraction in common below 80%.

### Samples included in RNAseq analysis

After all sample screening steps including quality of isolated RNA, success of sequencing and alignment, library quality, and confirmation of sample identity, the number of samples per group used for differential gene expression and WGCNA network analysis were: for AT, pre – 10, post – 9, washout – 8; and for SM, pre – 6, post – 8, washout – 7.

### Confounding factors check

Principle component analysis was run on AT and SM samples separately and PC1, PC2, and PC3 were evaluated for association with technical and phenotypical characteristics of the samples (RNA isolation and sequencing batches, sex, and starting body weight). None of these factors appeared to be major sources of variation among samples; samples instead group by treatment time point, except for the post-treatment SM samples (supplemental figure S6a-d). Additionally, degree of weight loss and starting body weight were not significantly correlated (figure S6e).

### RNAseq statistical analysis

Analysis of RNAseq expression data was performed using version 3.4.1 of the R programming language. Genes that had counts per million of at least 0.5 in one tenth of libraries were included in the analysis. In adipose tissue samples, 13,331 genes met these criteria and were included in the analysis. For muscle samples, 12,664 genes were included in the analysis. Gene expression counts were normalized within each tissue type with edgeR^72^ using the TMM method and voomWithQualityWeights from limma^73^. Differential gene expression analysis on individual genes was performed with limma and we considered an FDR < 0.05 to be a significant difference. Comparisons between treatment time points included time point and animal in the model to account for inter-animal differences. To test associations between change in gene expression and change in bodyweight, a general linear model in base R was used (difference in log2 gene expression between post and pre ~ percent change in body weight). P values were adjusted for multiple testing by the Benjamini-Hochberg false discovery rate (FDR) method, and a cutoff of FDR < 0.25 was used for significance. To find genes that were DE between pre- and post-treatment time points and whose change was associated with change in bodyweight, we included genes with an FDR < 0.25 in both the pre vs post and association with change in bodyweight comparisons.

Gene ontology and pathway analysis was performed using a hypergeometric p value test (base R phyper) to compare top differentially expressed (DE) genes to gene sets from the MSigDB C2 Canonical Pathways, and GO: Biological Processes collections^30^, as well as a curated list of obesity related gene sets downloaded from MSigDB, Ingenuity Pathway Analysis^22^ and relevant publications^13^ (supplemental table 7). From the post- vs pre-treatment comparison in AT samples, the top 100, 500, 1000, or all DE genes (2,005) with FDR < 0.05 and logFC> 0.26 or < -0.26 (20% change) were used. For genes whose expression was associated with both treatment and weight loss, the top 100, 500 or all DE genes (622) with an FDR < 0.25 and> 20% fold change in the pre- vs post-treatment comparison and an FDR < 0.25 in association with percent change in body weight were used. P values were adjusted for the number of gene sets tested with the FDR method.

Data-driven gene modules were constructed using Weighted Gene Correlation Network Analysis (WGCNA) version 1.68^11^ on samples from adipose tissue (27 samples) and skeletal muscle (21 samples) separately. Modules with a correlation of greater than 0.9 were merged, resulting in 71 modules for AT and 44 for SM. Modules were named AT_1-71 (adipose tissue) and SM_1-44 (skeletal muscle) after ordering the modules from greatest number of genes to smallest number of genes. Modules were annotated with gene sets used for pathway enrichment of top DEG using a hypergeometric test (base R phyper). P values were adjusted for the number of gene sets tested with the FDR method.

Gene module network plots were built using the igraph R package (igraph.org/r). The correlation between module eigengenes was calculated using base R cor.test, Spearman method. Correlations with a p value less than 0.05 and correlations stronger than 0.6 or -0.6 are included in Fig. 2 and Figure S2. For visual clarity, only modules significantly annotated with pathways related to metabolic disease were included.

Modules were tested for differential expression between treatment time points using a paired Wilcoxon test. A linear model was used to test for association between change in module expression and change in body weight. All p values were corrected by the FDR method for the number of modules tested, and a cutoff of FDR < 0.25 was used for significance

### *In vivo* mouse studies

#### Housing and diet

Eight to ten week-old male C57BL/6 N mice from Vital River Laboratories, China (or FAM19A4 and ANGPTL8) or Jackson Laboratories, USA (for NRG4) were used for gene overexpression studies. Mice were housed under conditions of 12 h light/dark cycle, controlled temperature (23 ± 2 °C), controlled humidity (55 ± 10%) and free access to water and food. Mice were single-housed as two mice in a cage with a divider, and were allowed to acclimate to single housing for one week prior to hydrodynamic gene delivery (HGD) procedure. Lean mice on a normal chow diet were used for FAM19A4 and ANGPTL8. For NRG4 studies in the DIO mouse model, the mice were fed with high-fat diet (60% calories from fat; D12492, Research Diets, New Brunswick, NJ) for 10 weeks from a starting age of 4 weeks old. All murine studies were approved by and carried out in accordance with IACUC guidelines at Novo Nordisk Research Center China.

#### Plasmids

Plasmids used to overexpress three targets (ANGPLT8, FAM19A4, and NRG4) were made by Genscript (Piscataway, NJ). In-house QC by kinetic turbidimetric assay with limulus amebocyte lysate was performed to ensure <5EU/kg body weight/mouse.

#### Hydrodynamic gene delivery (HGD)

Briefly, 2–2.5 mL of pre-warmed plasmid diluted in PBS were carefully injected into restrained, conscious mice via the tail vein within 5 seconds using a 25 G syringe. ANGPLT8, FAM19A4, NRG4, FGF21, and empty plasmids were given at 50 ug/mouse; Exendin-4 plasmid was given at 10 ug/mouse. Eight mice were used per treatment group.

#### ICV testing of FAM19A4

Mouse FAM19A4 (Uniprot Q7TPG5_S30-R135) was expressed in HEK 293 cells and purified by affinity and size exclusion chromatographies. The protein was formulated in 10 mM sodium phosphate, 161mM L-arginine, pH 7. The recombinant FAM19A4 protein carries an N-terminal glycine and proline, resulting from protease HRV 3 C cleavage of the protein from its expression fusion partner. The identity, purity, and stability of the FAM19A4 protein were confirmed by a variety of chemical and biophysical techniques. Mice were stereotaxically implanted with an intracerebroventricular cannula (Plastics One, Roanoke, VA) targeting the left lateral ventricle. To ensure proper cannula placement, NPY (5 ug diluted in PBS), an orexigenic peptide, was administered during the middle of the light cycle and subsequent food intake was measured. Mice that had failed to eat at least 0.4 g were excluded. Seven mice per group were given PBS, vehicle (10 mM PO4, 161mM L-arginine, pH7), or FAM19A4 protein. On testing days, food access was restricted and the animals were removed from their cages approximately 1 hour prior to lights off and injected with 2uL of either vehicle, PBS, or test compound. Once the last animal was injected, food access was restored.

#### Phenotypic and physiological measurements

Body weight and food intake were measured daily during HGD experiments. For ICV treatments, body weight was measured once daily via conventional scale and food intake was recorded hourly using an automated monitoring system (BioDAQ, Research Diets). Plasma triglycerides were measured by auto biochemical analyzer Cobas C501 (Roche Diagnostics), with reagents purchased from Roche or Wako Chemicals (Richmond, VA). To assess blood glucose, blood was collected from the tail vein into 5ul heparinized capillary tubes, immediately suspended in EKF system solution, and analyzed by glucose oxidase (BIOSEN 5040). BAT temperature was measured via a temperature transponder (IPTT-300, BMDS) implanted in the space above the interscapular region. Temperature readings were then obtained at the respective time points using a handheld scanner. Animals remained in their home cages during all temperature readings.

#### Statistical analyses

Differences between treatment groups in mouse studies were evaluated using one-way ANOVA with Dunnett’s post-hoc test for multiple comparisons. Total change in body weight between FAM19A4 and vehicle and change in BAT between baseline and post-injection in the FAM19A4 ICV experiment were assessed by two-tailed T-test.

## Supplementary information

Supplementary figures.

Supplementary dataset.

## Data Availability

The FGF21-treated NHP RNAseq data generated during and analyzed for the current study are available in the NCBI GEO repository, accession number GSE134213: https://www.ncbi.nlm.nih.gov/geo/query/acc.cgi?acc=GSE134213 The R programming code used to analyze the RNAseq data set is available on GitHub: https://github.com/smurray33/FGF21_NHP_RNAseq.
