# Supplementary material for: Whole transcriptome analysis and validation of metabolic pathways in subcutaneous adipose tissues during FGF21-induced weight loss in non-human primates
Source: Sci Rep. 2020 Apr 29;10:7287. doi: 10.1038/s41598-020-64170-6 (PMC7190698; doi:10.1038/s41598-020-64170-6)
Supplement: Supplementary file 1 — Supplementary figures. [file 41598_2020_64170_MOESM1_ESM.pdf]

Supplementary Figures

Whole transcriptome analysis and validation of metabolic pathways in subcutaneous adipose tissues during FGF21-induced weight loss in non-human primates

Sara A. Murray, Louise S. Dalbøge, Karalee Baquero, Christina A. Sanford, Ayesha Misquith, Aaron J. Mercer, Thomas H. Meek, Mette Guldbrandt, Birgitte Andersen, Paul Kievit, Kevin L. Grove, Burak Kutlu

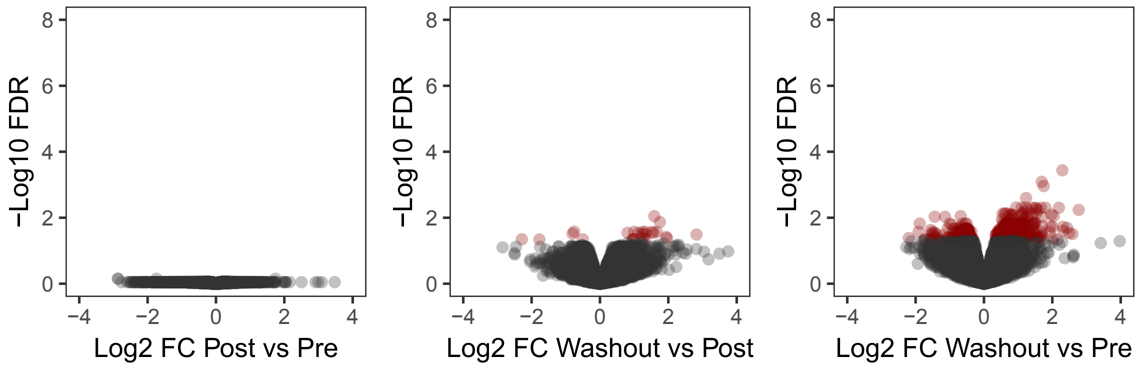

Figure S1) FGF21 treatment and weight loss-associated transcriptional changes in skeletal muscle. Gene expression in gastrocnemius muscle was measured by RNA sequencing. Fold change (x axis) and statistical significance (y axis) of genes differentially expressed between treatment time points. Genes with fold change > 0.26 or < -0.26 and FDR < 0.05 are highlighted in red.

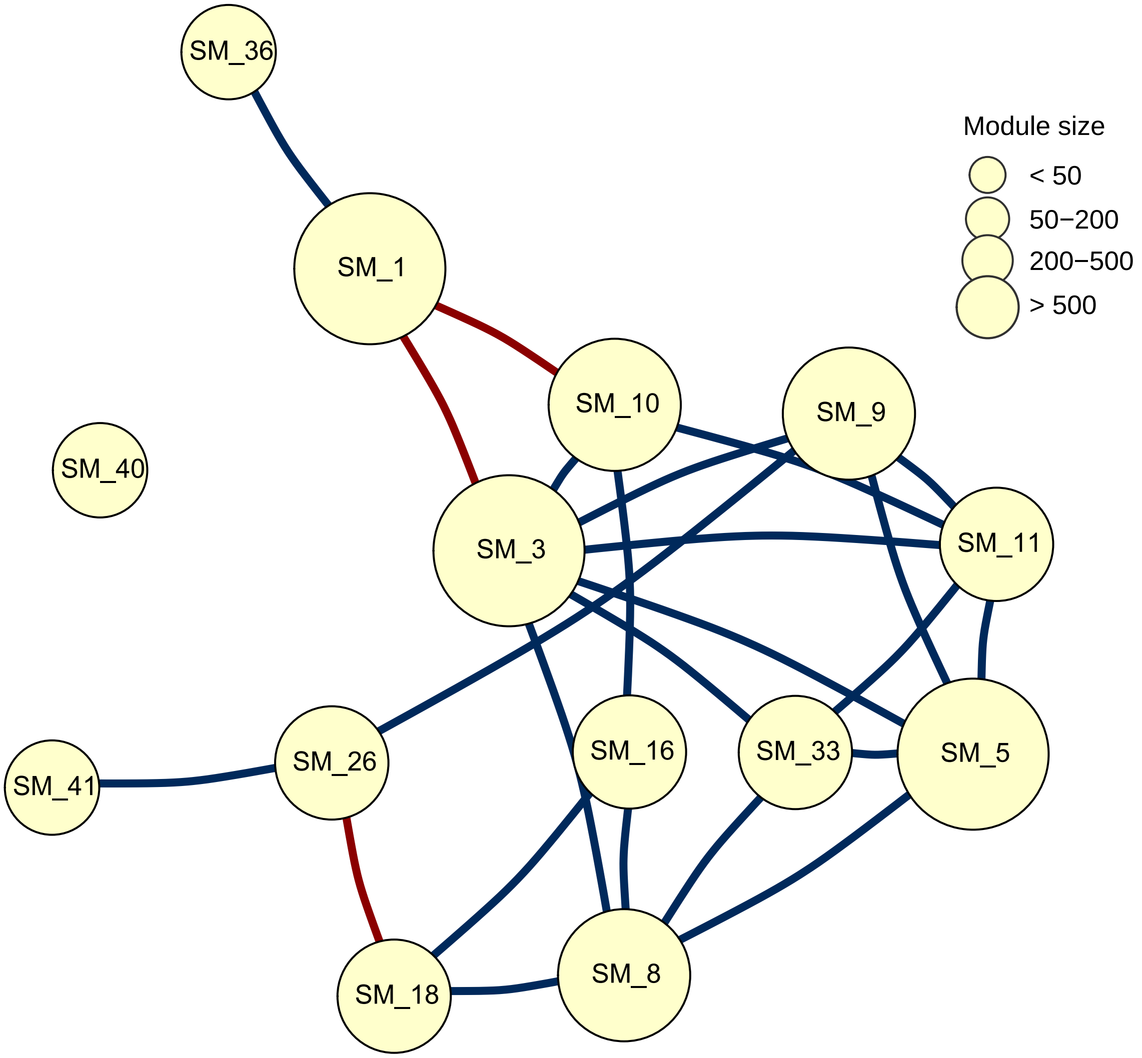

Module	Annotation summary	Module	Annotation summary
SM_1	Vasculogenesis, extracellular matrix	SM_16	Oxidative phosphorylation, mitochondria
SM_3	Glycolysis, gluconeogenesis	SM_18	Oxidative phosphorylation, mitochondria
SM_5	Stress response	SM_26	Vasculogenesis
SM_8	Insulin signaling	SM_33	Hypoxia
SM_9	Stress response	SM_36	Lipoprotein clearance, insulin resistance
SM_10	Oxidative phosphorylation, mitochondria	SM_40	Myogenesis
SM_11	Hypoxia	SM_41	Vasculogenesis

Figure S2. Correlation network of metabolism-related SM gene modules. As in Figure 2, WGCNA was used to group genes into modules within SM. Nodes represent gene modules and edges represent Spearman correlation between module eigengenes. Edge color represents positive (blue) or negative (red) correlation between module expression. Only correlations stronger than r = 0.6 or -0.6 are shown. Node size depicts the number of genes in the module. No SM gene modules were statistically significantly associated with change in body weight. Bottom, short descriptions of each module included in the network, based on overlap with known biological pathways (see supplemental table S3 for full list of annotations).

a

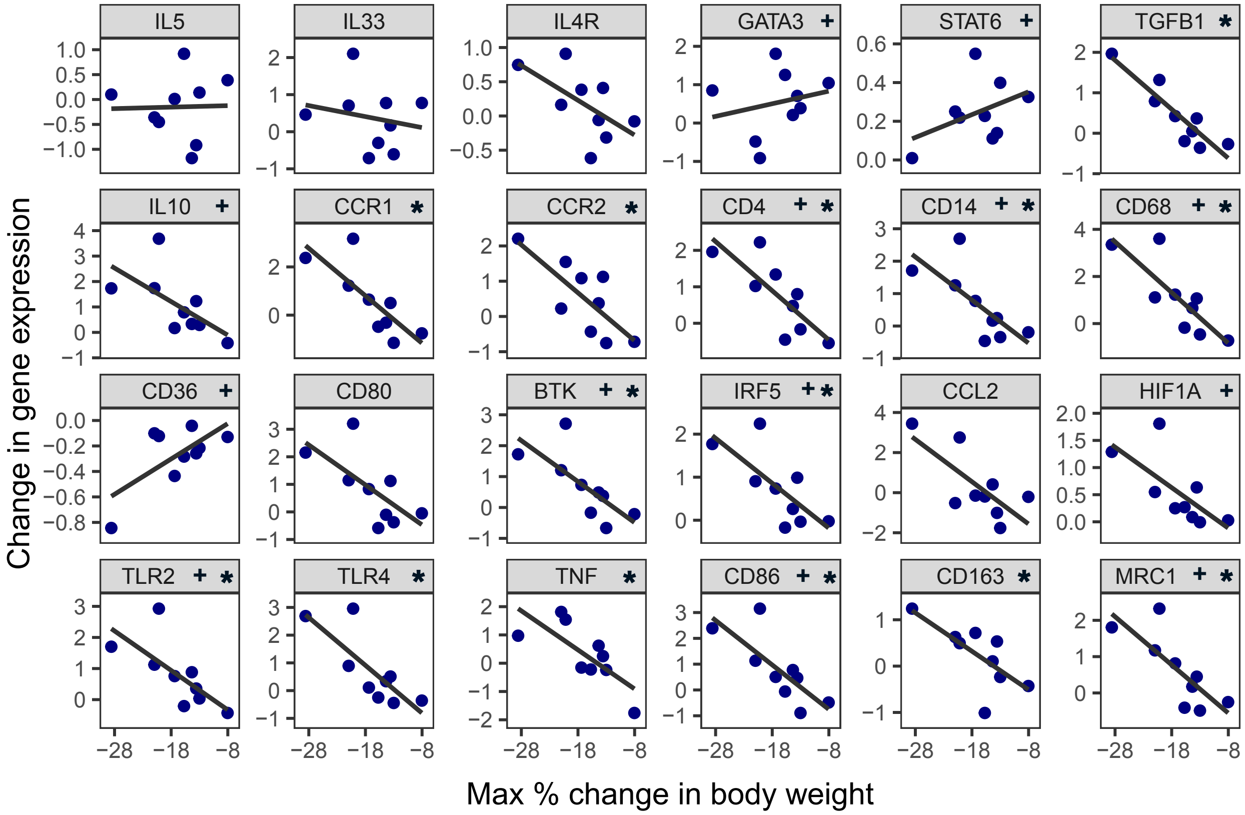

Figure S3) Change in expression of metabolism, neuronal, and immune genes and their association with change in body weight. Genes from Figure 4 are plotted individually as change in gene expression between pre and post time points against change in body weight between pre and post time points. A) Genes related to type 2 immune signaling and innate immune cells. B) (Next page) Genes involved in thermogenesis, lipid storage, neuronal signaling. +, genes with significantly different expression between pre and post time points (FDR < 0.05); *, genes whose change in expression was significantly associated with decreased body (FDR < 0.25).

Figure S3

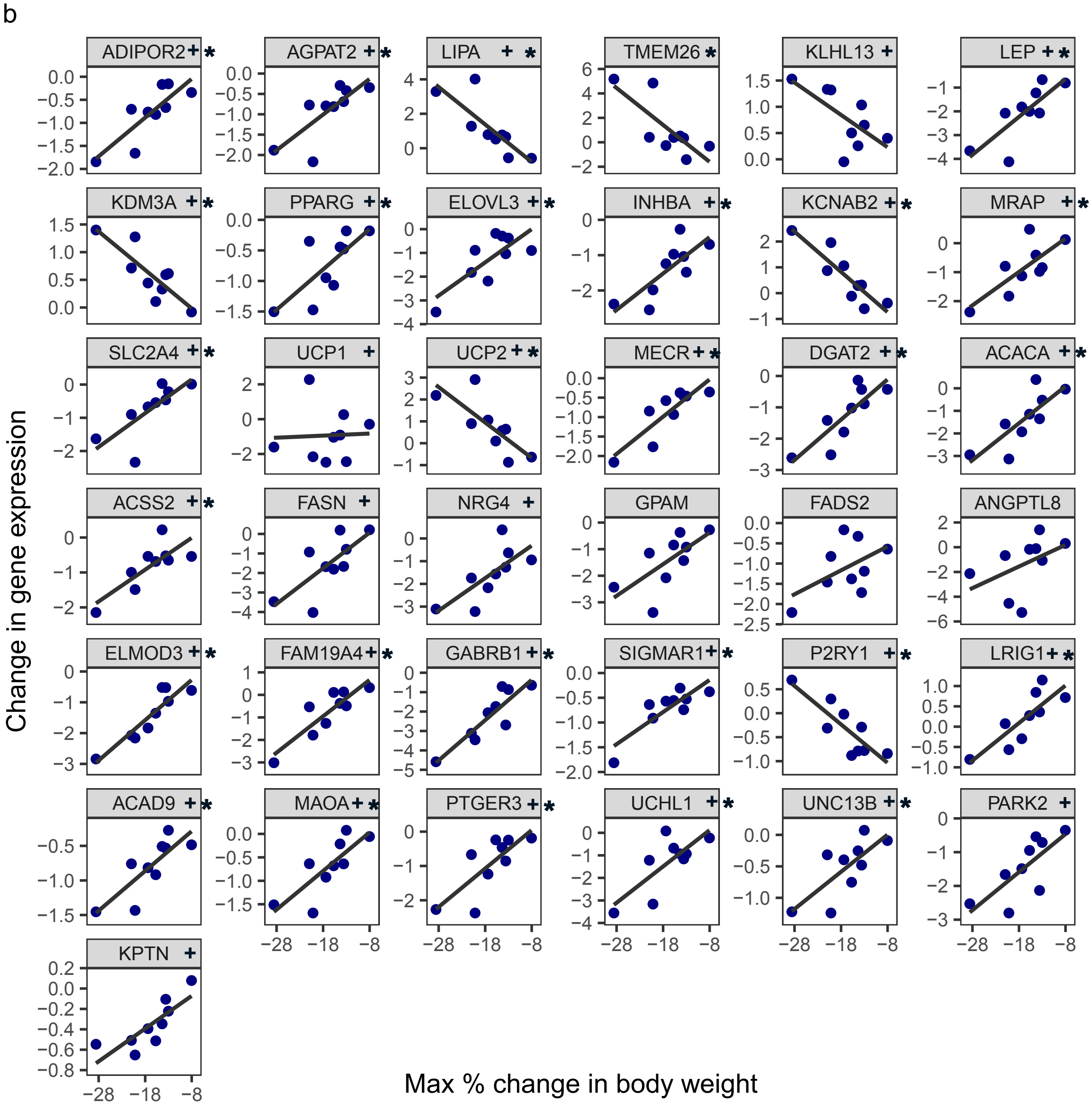
b
ADIPOR2 + *
AGPAT2 + *
LIPA + *
TMEM26 *
KLHL13 +
LEP + *
KDM3A + *
PPARG + *
ELOVL3 + *
INHBA + *
KCNAB2 + *
MRAP + *
SLC2A4 + *
UCP1 +
UCP2 + *
MECR + *
DGAT2 + *
ACACA + *
ACSS2 + *
FASN +
NRG4 +
GPAM
FADS2
ANGPTL8
ELMOD3 + *
FAM19A4 + *
GABRB1 + *
SIGMAR1 + *
P2RY1 + *
LRIG1 + *
ACAD9 + *
MAOA + *
PTGER3 + *
UCHL1 + *
UNC13B + *
PARK2 +
KPTN +
Change in gene expression
Max % change in body weight

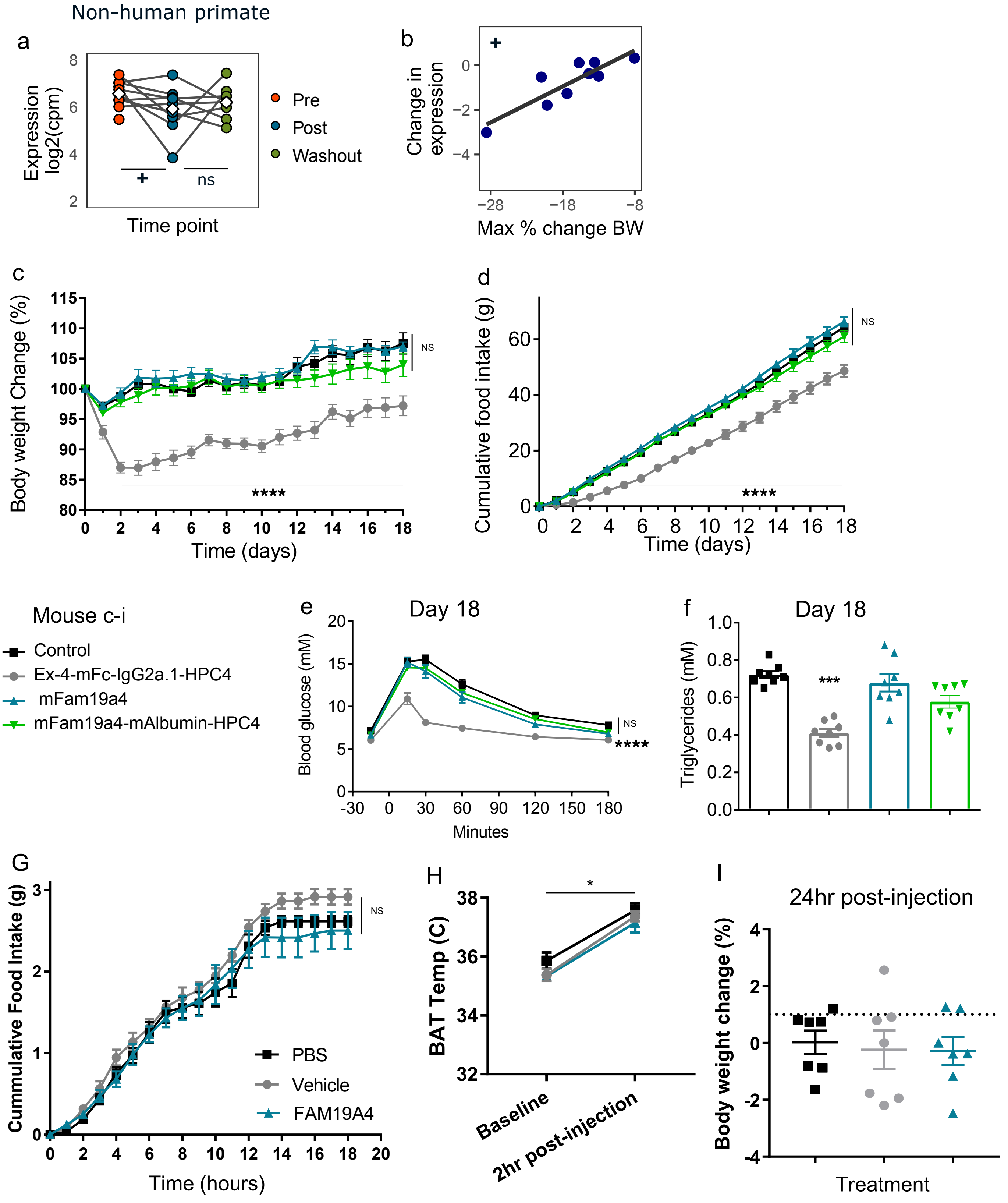

Figure S4) FAM19A4 treatment has no effect on body weight in mice. FAM19A4 was overexpressed in mice by hydrodynamic gene delivery. A) Expression levels and B) association with change in body weight in FGF21-treated NHP. C-F) Mice were given empty plasmid, Exendin-4, native mouse Fam19a4, or Fam19A4 fused to mouse albumin. C) Change in body weight and D) cumulative food intake over time E) Blood glucose after glucose tolerance test and F) plasma triglycerides at day 14. G-I) Mice were outfitted with intracerebral ventricle cannulas and treated with PBS, vehicle, or Fam19A4 protein. G) Cumulative food intake over 24hrs post-treatment. H) Brown Adipose Tissue (BAT) temperature 2hrs post-treatment. I) Change in body weight 24hrs post-treatment. Statistical notations: NS non-significant; A-B) + FDR < 0.25 by linear model; C-F) Data are displayed as mean plus standard error, n=7-8 per group, * p < 0.05, ** p < 0.01, *** p < 0.001 by one-way ANOVA with Dunnett's post-hoc test (C-G) or two-tailed T-test (H-I).

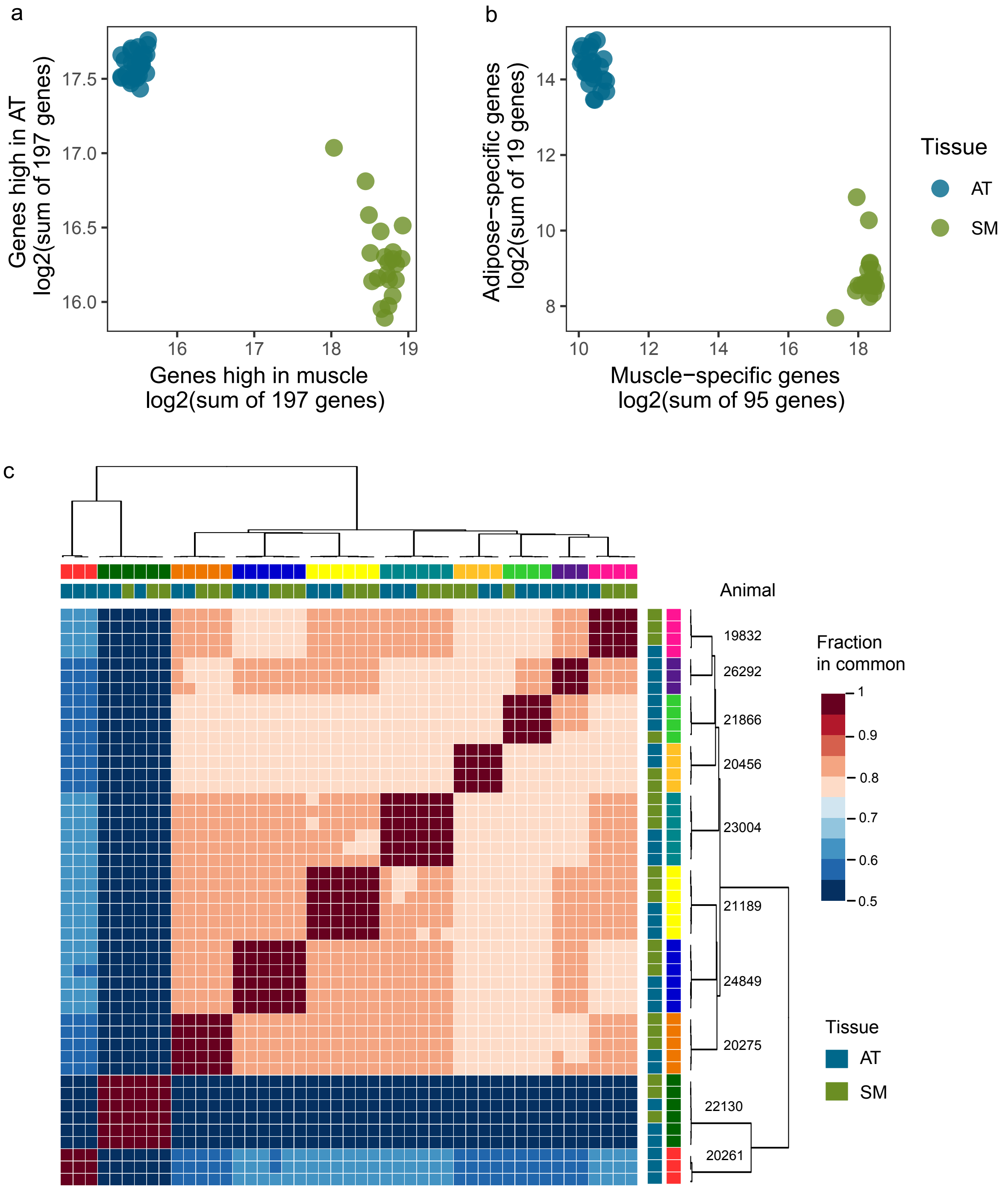

Figure S5) Sample identity was confirmed using tissue specific genes and genomic variants. The correct identity of RNAseq samples was confirmed computationally. Using gene expression data from the Human Protein Atlas[69], all samples were plotted by expression of genes that were A) among the 1% most highly expressed in either AT or SM, or B) expressed at 5-fold greater levels in AT or SM compared to all other tissues assayed. C) Hierarchically clustered heatmap depicting the fraction of genomic variants in common between two samples, out of 5,000 loci that were highly expressed across all samples. The similarity of samples from each individual animal (colored bars on top and right edge) from different tissues (green, SM; and blue, AT) and time points is depicted by the dendrograms above and to the right of the heatmap.

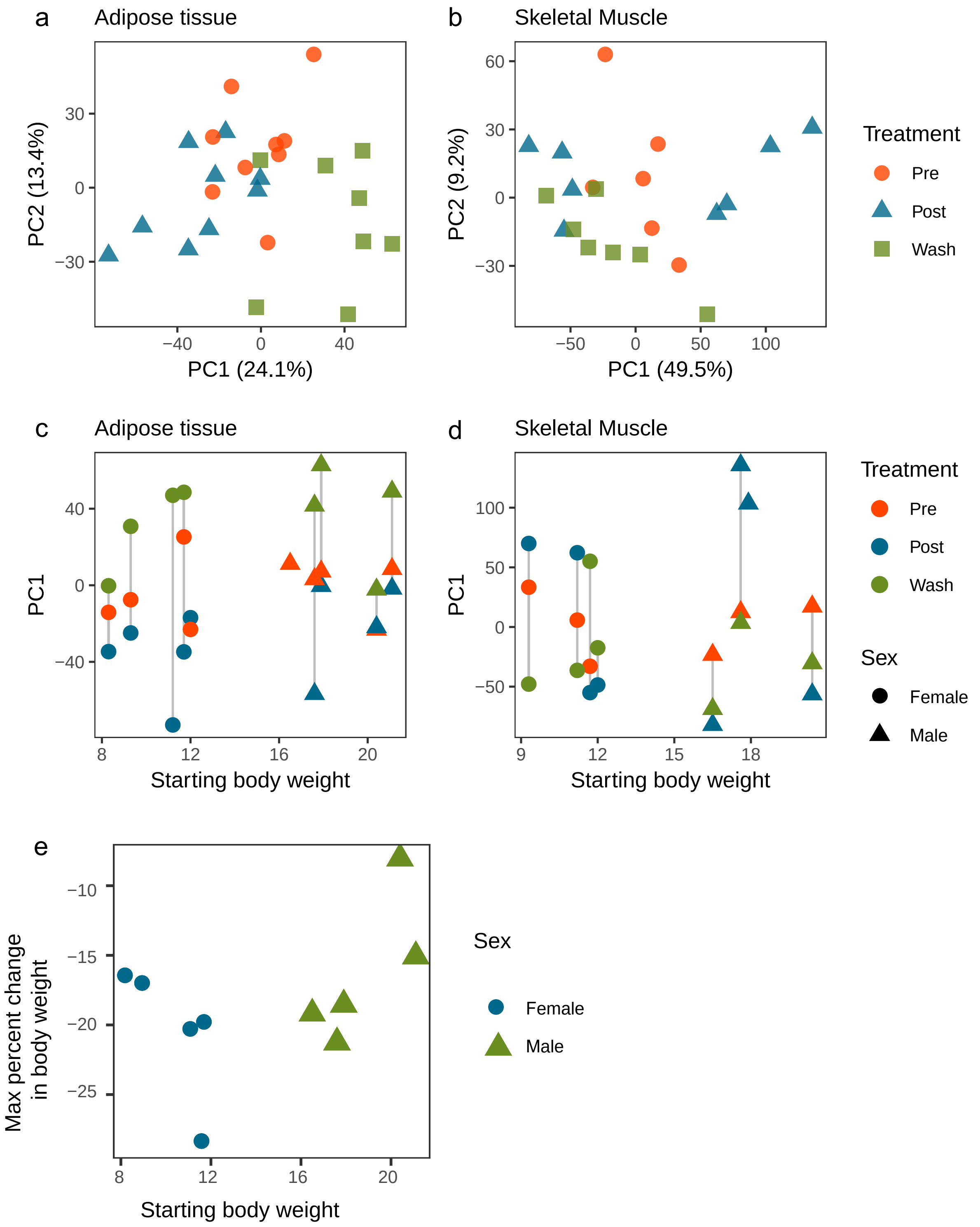

Figure S6) Results were checked for confounding factors with principle component analysis. PCA plots colored by treatment time point for A) AT and B) SM. Starting body weight plotted against PC1 for C) AT and D) SM. Samples from the same animal are connected with grey lines, and sex is indicated by point shape. E) Maximum percent change in body weight was not statistically significantly correlated to starting body weight (all animals together) by Spearman's rank correlation (p =.11, rho = 32)
